# Exaggerated perception of change with greater sensory imprecision

**DOI:** 10.1038/s41598-025-14791-6

**Published:** 2025-08-11

**Authors:** George T. Herbert, Choi Tim Ho, Julia Goddard, Harry J. Garthwaite, Jessica Komes, Christopher I. Petkov, William Sedley

**Affiliations:** 1 Translational and Clinical Research Institute, Newcastle University, Newcastle upon Tyne, NE2 4HH UK; 2 School of Psychology, Newcastle University, Newcastle upon Tyne, NE2 4HH UK; 3 Department of Cognitive Disorders and Old Age Psychiatry, Department of Medical Psychology & Psychiatry, University Hospital of Bonn, Venusberg Campus 1, 53127 Bonn, Germany; 4 Department of Neurosurgery, University of Iowa, 1800 JPP, 200 Hawkins Drive, Iowa City, IA 52242 USA

**Keywords:** Perceptual inference, Stimulus precision, Bayesian inference, Perception, Human behaviour, Sensory processing, Perception

## Abstract

Bayesian models describe precision (inverse variance) as a key determinant of perception. However, there is limited evidence on the behavioural effects of precision. The default assumption is that higher precision leads to greater surprise (or perceived change) from otherwise equivalent sensory changes. Four human experiments investigated the influence of precision on perceived salience of systematic changes in auditory stimulus streams. Participants reported Perceived Salience of Change (PSC) in the mean value of Gaussian sequences of pure tones varying in either frequency or intensity, with sequences differing in precision. We hypothesised that PSC, for a particular absolute mean change, would positively correlate with stimulus precision. Surprisingly, we observed multiple instances of the opposite effect, where PSC was rated as higher in low-precision conditions. The conditions under which we found evidence for a counter-Bayesian strategy was under extreme values of individual stimuli within sequences, and mostly in experiments where frequency rather than intensity was the varied parameter. Further scrutiny of the specific conditions for these surprising results showed that low precision could be associated with worsened, unaffected or improved correct reporting of the direction of sound frequency change. These results raise the intriguing possibility that certain circumstances, particularly those characterised by low signal-to-noise, human perception may adopt a counter-Bayesian strategy, and we discuss the potential mechanisms, evolutionary benefits, and clinical implications for future work to further test this falsifiable hypothesis.

## Introduction

Contrary to classical theories of sensory processing, perception is now broadly considered an active process, where incoming sensory information is used to update internal models of the environment^1^. Predictive coding, initially proposed by Rao and Ballard^2^, is one such generative account, describing how the brain functions as a Bayesian inference machine, constantly generating and updating an internal model of the environment. Various frameworks exist, with many similarities, such as those falling under the umbrellas of *active sensing*^3^ and the *free energy principle*^4,5^.

A fundamental principle common to these accounts is the optimisation of inference under conditions of uncertainty; perception results from an interplay of internal representations of the environment (*priors*, which generate *predictions*) combined with incoming sensory input (likelihood – i.e. the *likelihood* that the pattern of sensory input corresponds to a particular environmental state or object). To optimise this balance, prior predictions and sensory input are each weighted by their *precision*. Incongruence between prior predictions and sensory input is termed *prediction error* (mismatch between prior and likelihood), also known as *surprise*. It is widely assumed that subjective and objective surprise responses are proportional to both the precision of the likelihood (sometimes termed *precision-weighted prediction error – PWPE*), and the precision of the prior (with the term *surprise* referring to the improbability of the sensory input based on the prior). Surprise is mathematically defined as the negative log probability of the sensory input according to the prior, and this accords well with the lay intuitive notion of surprise as the cognitive/affective sense of the unexpectedness of an event or stimulus.

In terms of predictive processing, therefore, perception is the act of minimising surprise through continuous updating of internal models. As well as surprise (as in prediction error), another important quantity in this process is *Bayesian surprise*, which is the extent to which the internal model is updated on account of the stimulus (i.e. the difference between the posterior and the prior). As with surprise more generally, Bayesian surprise has both subjective^6^ and objective correlates^7^.

Mathematically, precision is the inverse of variance. In the brain, *estimated precision* is encoded largely by postsynaptic gain^8,9^. ‘Precision’ thus refers to two related but distinct phenomena: the statistical properties of the sensory input from the environment, and the brain’s estimate of the reliability or importance of the signal from the environment. It is often assumed that these two phenomena are closely correlated. However, if there are systematic ways in which the brain’s estimated precision deviates from environmental precision, this could have important implications in understanding neurotypical, atypical and disordered brain function. A related general assumption is that higher sensory precision is associated with greater perceptual salience of a change than the same change under lower precision conditions. However, to our knowledge this has not been directly tested.

**Fig. 1:**
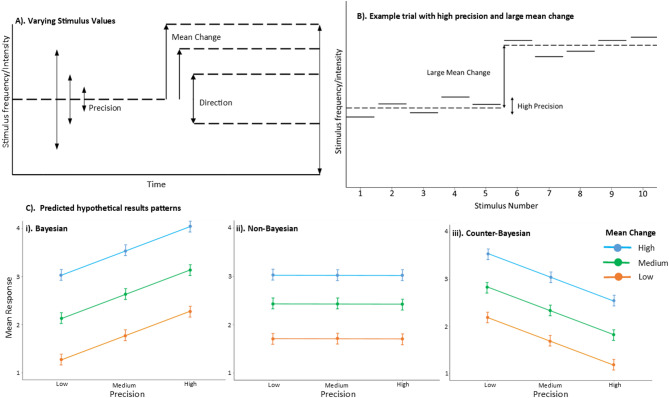
Overview of experimental paradigm, and canonical predicted results patterns. **(A)** Varying experimental stimulus parameters. Low, medium, and high precision (long, medium and short, respectively) and mean change values are indicated by arrows. Direction indicates whether the mean change was either an increase or a decrease in either frequency or intensity. **(B)** Example trial with high precision and high mean change in upward direction. Each solid horizontal line indicates one stimulus. Dashed lines indicate the mean of the Gaussian distribution from which stimuli are drawn. **(C)** Predicted results patterns based on three hypothesised canonical relationships between stimulus precision and perceived salience of change (note: direction of mean change not specified): **i)**. Bayesian, or signal-to-noise ratio (preferred); **ii)** Non-Bayesian, or precision-indifferent (possible); **iii)** Counter-Bayesian (not anticipated). Note: combinations of response patterns might also be seen.

This study describes the implementation of a behavioural auditory Bayesian surprise paradigm (Fig. 1a & b), over a series of several experiments (Exp. 1 to 4), to explore intrinsic bias in how stimulus sequence properties (principally precision, i.e. inverse variance of the distributions from which stimuli are drawn) influence the perception of otherwise equivalent sensory changes. We specifically examine the perception of, and subjective response to, a systematic change in the underlying stimulus distribution, i.e. Bayesian surprise, rather than the surprise associated with individual stimuli. Hereafter we use the term ‘perceived salience of change (PSC)’ to refer to this subjective sense of Bayesian surprise. Subjects listen to sequences of pure tones drawn from Gaussian distributions with a specified mean and precision (i.e. inverse variance) (Fig. 1a), in which a change in mean tone frequency (or intensity in Exp. 4) of the distribution occurs exactly half-way through the sequence (Fig. 1b), and subsequently provide ratings of PSC. In all studies reported here, the principal dependent variable was PSC, on a Likert scale of 1–4, i.e. reported sense of how much the mean of the Gaussian distribution, from which stimuli are drawn, changed during the trial. Exp. 1 asked participants how ‘noticeable’ they found the change in mean stimuli, and Exp. 2–4, asked how ‘surprising’ they found this change. Exp. 3 altered the timescale of the stimuli, having a larger number of shorter stimuli in each trial. In Exp. 4 the stimuli varied in intensity rather than frequency. Exp. 3 and 4 additionally asked participants to report the direction of mean change as well as reported surprise, to obtain a behavioural performance measure.

The aim of these experiments was to investigate the relationship between changing statistical parameters of the auditory stimuli and this PSC, particularly the effect of stimulus precision, and the interaction of precision and mean change. As greater stimulus precision would increase the signal-to-noise ratio and allow more accurate estimation of the mean stimulus change, we predicted, in all four experiments, that PSC would increase with greater stimulus precision, as well as with greater absolute mean change. This relationship would indicate a Bayes-optimal response, which would be evident in a positive linear relationship between mean PSC response and precision, as shown in Fig. 1C(i). A non-Bayesian response pattern would show no relationship between mean response and precision (Fig. 1Cii) and would indicate that the extra noise added by reduced stimulus precision did not affect participants perception of the salience of, or ability to detect, stimulus changes. A counter-Bayesian response pattern would be indicated by a negative linear relationship between precision and PSC (1Ciii). Although we did not expect evidence for counter-Bayesian human behaviour, once we observed evidence for it with the first experiment, we initiated several other experiments to scrutinize the conditions under which counter-Bayesian human behaviour may occur. We found that certain low signal-to-noise conditions could elicit counter-Bayesian behaviour in the human participants.]

## Results

Participants in all four experiments utilised the full range of perceived salience of change values in their responses, which ranged from 1 to 4. Means (and standard deviations) of all response values from Exp. 1, 2, 3, and 4, were respectively: 2.66 (± 0.70), 1.87 (± 0.69), 2.05 (± 0.62), 2.03 (± 0.68). Mean accuracy measures from Exp. 3 and 4, were 0.79 (± 0.20), and 0.74 (± 0.23), respectively (with chance level being 0.5).

**Fig. 2:**
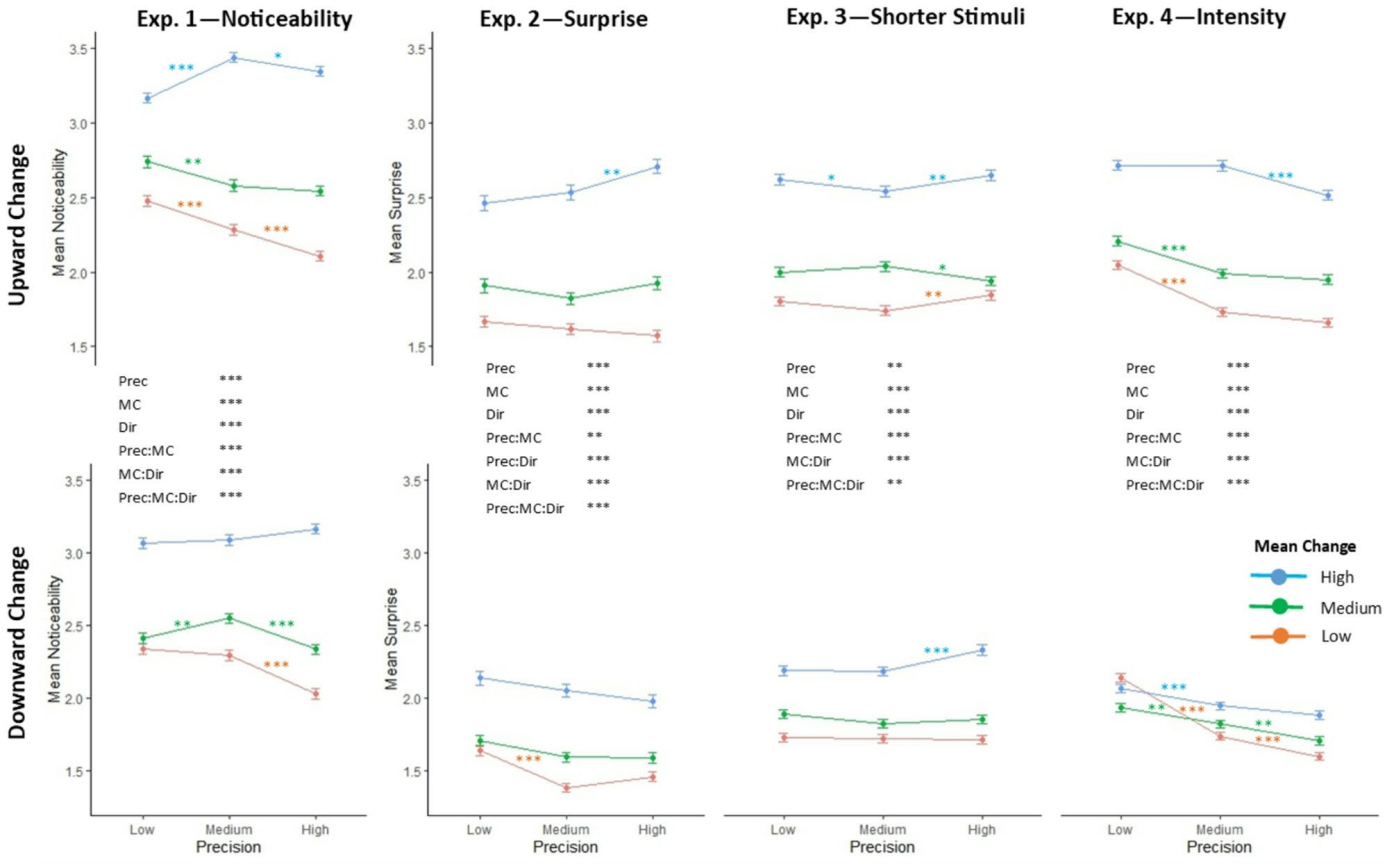
Perceived salience of mean change (PSC) response patterns for all experiments. Mean and standard error of PSC ratings across participant group for each stimulus category, from Experiments 1, 2, 3, and 4 in their respective columns. Y axis labels ‘noticeability’ and ‘surprise’ refer to what participants were asked to rate to indicate PSC. The x axis indicates precision of stimulus sequence, colour indicates salience of mean change (response scale range 1–4). The row indicates the direction of the mean change (in frequency or intensity): top row = upward, bottom row = downward. Significant main effects and interaction effects are shown in black text for each experiment. Shorthand for stimulus parameters are as follows: Prec = stimulus precision, MC = mean change size, Dir = direction of the mean change. Coloured asterisks indicate statistical significance between neighbouring precision values for each stimulus-mean-change combination based on pairwise post-hoc testing of significant main and interaction effects. Significance is indicated as * if *p* < 0.05, ** if *p* < 0.01, and *** if *p* < 0.001.

Mean PSC response increased with greater mean change (Fig. 2). However, contrary to the Bayesian hypothesis (Fig. 1Ci), PSC did not appear to consistently increase with stimulus precision (Fig. 2). Experiments 1, 2, and 4 had mean change conditions in which higher precision resulted in lower PSC, which indicates a counter-Bayesian response pattern (Fig. 1Ciii). Results are described more fully in their respective experiment sections below, the full breakdown of all interaction effects can be found in the OSF file linked in the data availability statement. In Exp. 1 and 2, generally, larger mean change conditions were associated with positive relationships between precision and subjective change (Bayesian response pattern), whilst smaller mean changes were associated with an inverse relationship (counter-Bayesian response pattern). Exp. 4, where stimulus intensity rather than frequency was the varied parameter, showed only the counter-Bayesian pattern. Exp. 3, featuring a larger number of shorter stimuli, had effects of precision that were neither simply positive or negative correlations, but showed more complicated relationships, characterised by non-Bayesian or mixed response patterns (Fig. 1Cii).

**Fig. 3:**
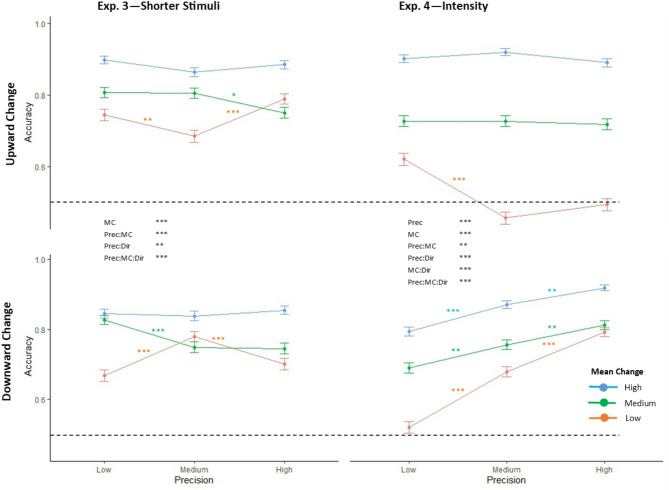
Accuracy of identifying direction of mean change. Mean and standard error of accuracy (proportion of mean change values correctly identified as increases or decreases in frequency/intensity) values for Exp. 3 and Exp. 4. The x axis indicates precision, colour indicates mean change. Top row indicates mean increases (in frequency/intensity), and bottom row mean decreases. Dashed line indicates chance level (0.5). Significant main effects and interaction effects are shown in black text for each experiment. Shorthand for stimulus parameters are as follows: Prec = stimulus precision, MC = mean change size, Dir = direction of the mean change. Coloured asterisks indicate significant differences between neighbouring precision values for each stimulus condition, based on post-hoc pairwise testing of significant main and interaction effects. Significance is indicated as * if *p* < 0.05, ** if *p* < 0.01, and *** if *p* < 0.001.

### Experiment 1: ‘Noticeability’ as subjective response

A three-way repeated measures ANOVA on subjective change response showed significant main effects of precision (F(1.8, 131.52) = 26.965, *p* < 0.001), mean change (F(1.1, 80.26) = 191.220, *p* < 0.001), and direction (F(1,73) = 35.665, *p* < 0.001). Significant interaction effects were shown between: precision and mean change (F(4, 292) = 33.531, *p* < 0.001); mean change and direction (F(2, 146) = 9.808, *p* < 0.001); and mean change, precision, and direction (F(4, 292) = 12.794, *p* < 0.001). PSC was greater for larger mean changes and for upward changes of frequency. PSC did not increase with precision in the predicted Bayesian response pattern(1Ci); with medium and low mean change size, PSC decreased with increased precision, in a counter-Bayesian manner. With a high mean change size for downward frequency changes, the results indicated a non-Bayesian response pattern (Fig. 1Cii). The high mean change for upward frequency changes seemed to indicate conflicting patterns, with medium precision giving the highest PSC response.

### Experiment 2: ‘Surprise’ as subjective response

We observed a significant effect of precision (F(2, 94) = 8.405, *p* < 0.001); mean change (F(1.14, 53.6) = 118.716, *p* < 0.001); and direction (F(1, 47) = 50.716, *p* < 0.001). Interaction effects were found between: precision and mean change (F(4, 188) = 4.208, *p* = 0.003); precision and direction (F(2, 94) = 17.556, *p* < 0.001); mean change and direction (F(1.48, 69.6) = 32.078, *p* < 0.001); and precision, mean change, and direction (F(4, 188) = 7.767, *p* < 0.001).

With downward changes, precision behaved solely in a counter-Bayesian manner, albeit generally not to a statistically significant degree (with the notable exception of low precision responses being higher than medium precision responses at a low mean change). Upward changes showed more complicated relationships. High mean change indicated a Bayesian response pattern: medium and low mean change indicated non-Bayesian response patterns (or at low mean change a trend towards counter-Bayesian response pattern).

### Experiment 3: Larger number of shorter stimuli

#### Subjective change responses

We observed significant effects of: precision (F(2, 154) = 6.109, *p* = 0.003); mean change (F(1.11, 85.75) = 130.075, *p* < 0.001); and direction (F(1,77) = 79.995, *p* < 0.001. Interaction effects were found between: precision and mean change (F(4, 308) = 5.580, *p* < 0.001); mean change and direction (F(2, 154) = 34.560, *p* < 0.001), and precision, mean change, and direction (F(4, 308) = 4.605, *p* = 0.001). Relationships between PSC and precision largely indicate a non-Bayesian response pattern, but with some instances of Bayesian and counter-Bayesian patterns between related stimulus conditions, e.g. with a high or low mean change, medium precision yielded the lowest response (i.e. mixed Bayesian and counter-Bayesian), whilst for medium mean change high precision gave the smallest PSC ratings (i.e. counter-Bayesian).

#### Accuracy of direction responses

We observed a significant effect of mean change (F(1.79, 137.73) = 100.019, *p* < 0.001). Interaction effects were found between: precision and mean change (F(3.41, 262.83) = 9.609, *p* < 0.001); precision and direction (F(2, 154) 5.121, *p* = 0.007); and precision, mean change, and direction (F(4, 308) = 14.118, *p* < 0.001). Relationships between accuracy of direction response and precision indicated conflicting response patterns, with some instances of Bayesian or counter-Bayesian patterns between related stimulus conditions (Fig. 3); e.g. In the low mean change condition, low and high precision biased towards reporting an upward frequency change (i.e. higher accuracy for upward change and lower accuracy for downward change), whilst medium precision favoured reporting a downward change. In a somewhat different pattern, for medium mean change, low precision gave the overall highest accuracy, and high precision the lowest accuracy, indicating a fully counter-Bayesian pattern (which coincided with the fully counter-Bayesian pattern seen in the PSC responses for these conditions). High mean change indicated a non-Bayesian response pattern, i.e. being unaffected by precision (Fig. 1Cii).

### Experiment 4: Intensity rather than frequency changes.

#### Subjective change responses

We observed effects of: precision (F(1.37, 128.91) = 90.050, *p* < 0.001), mean change (F(1.19, 111.61) = 119.075, *p* < 0.001), and direction (F(1, 94) = 124.812, p = < 0.001). Interaction effects were found between precision and mean change (F(4, 376) = 25.673, *p* < 0.001); mean change and direction (F(1.39, 130.91) = 125.923, *p* < 0.001); and precision, mean change, and direction (F(4, 376) = 7.558, *p* < 0.001). Relationships between PSC and precision largely indicate a counter-Bayesian response pattern (Fig. 1ciii), with greater PSC at lower levels of precision (Fig. 2).

#### Accuracy of direction responses

We observed effects of precision (F(2, 188) = 39.306, *p* < 0.001), and mean change (F(1.69, 158.91) = 563.735, *p* < 0.001). Interaction effects were found between: precision and mean change (F(3.44, 323.41) = 4.319, *p* = 0.003); precision and direction (F(1.84, 173.29) = 92.633, *p* < 0.001), mean change and direction (F(1.78, 167.43) = 34.347, p = < 0.001); and precision, mean change, and direction (F(4, 376) = 23.731, *p* < 0.001). In the downward intensity change condition, relationships between accuracy of change response and precision indicated a Bayesian response pattern, with greater precision leading to greater accuracy (Fig. 3). In the upward intensity change condition, precision largely indicated non-Bayesian response pattern (Fig. 3). The low mean change condition deviated from this pattern, with significantly better accuracy for low precision than medium or high precision, which were both around 50% accuracy (chance level).

### Influence of extreme values on behavioural results

As summarised in Table 1, we observed effects of extreme values of individual stimuli within the trial, which depended upon the experiment, the direction of change, and the precision value. The strongest correlations were with the ‘min2-max1’ metric (i.e. lowest value in second half minus highest value in first half), followed by the ‘max2-min1’ metric (i.e. highest value in second half minus lowest value in first half), followed by change between the stimuli immediately either side of the half-way point. These were most influential for Exp. 2, with a total of 21 of the 54 condition-metric combinations showing uncorrected significant correlations, of which 6 survived FDR correction. Conversely, Exp. 3 and 4 showed only 2 and 3 significant uncorrected correlations (approximately chance level), respectively, of which none survived FDR correction. The ‘min2-max1’ metric showed significant correlations at all three precision values, whereas ‘max2-min1’ was more influential at lower precision values, and halfway change predominantly for mid-precision.

**Table 1:** Influence of extreme individual stimulus values within a trial on behavioural responses. The table summarises spearman correlation testing between three metrics of extreme individual stimulus values (defined below) and group mean behavioural PSC response, across the 10 exemplars in each stimulus category. ‘Stim. 6 − 5’ indicates the size of difference (in frequency for exp. 2 and 3, intensity in exp. 4) between the last stimulus in the first half of the stimuli and the first stimulus of the second half of the stimuli (stimulus 6 - stimulus 5 in exp. 2 and 4, stimulus 16 - stimulus 15 in exp. 3). ‘min2 - max1’ indicates the difference between the lowest value (frequency in exp. 2 and 3, intensity in exp. 3) of the second half of the stimuli and the highest value of the first half of the stimuli. Note that this value was usually negative. ‘max2 - min1’ reflects the difference between the highest value (frequency in exp. 2 and 3, intensity in exp. 4) of the second half of the stimuli and the lowest value of the first half of the stimuli. *ρ* (Rho) indicates the spearman correlation coefficient value. P. indicates the P.value. P-values < 0.05 are in **bold**, P.values < 0.05 FDR adjusted are in ***bold italics***. Prec. = P.ecision, MCh. = mean change, Dir. = direction of mean change.

			Exp. 2 (surprise)						Exp. 3 (timescale)						Exp. 4 (intensity)					
			Stim. 6 − 5		min2-max1		max2-min1		Stim. 6 − 5		min2-max1		max2-min1		Stim. 6 − 5		min2-max1		max2-min1	
Prec.	MCh.	Dir.	*ρ*	p.	*ρ*	p.	*ρ*	p.	*ρ*	p.	*ρ*	p.	*ρ*	p.	*ρ*	p.	*ρ*	p.	*ρ*	p.
low	low	dn.	− 0.07	0.14	− 0.14	**0.00**	− 0.03	0.55	− 0.41	0.24	− 0.68	**0.03**	− 0.42	0.23	0.27	0.45	− 0.32	0.37	0.03	0.93
low	med.	dn.	0.00	0.98	− 0.07	0.15	− 0.04	0.38	0.55	0.10	− 0.16	0.65	− 0.04	0.91	− 0.32	0.37	− 0.83	**0.00**	− 0.28	0.43
low	high	dn.	− 0.07	0.15	− 0.07	0.15	− 0.03	0.50	− 0.60	0.07	− 0.20	0.58	− 0.28	0.43	− 0.18	0.63	− 0.52	0.13	− 0.27	0.45
med.	low	dn.	− 0.03	0.56	− 0.07	0.14	− 0.06	0.16	− 0.13	0.72	− 0.53	0.11	0.10	0.77	− 0.29	0.41	− 0.28	0.43	0.25	0.49
med.	med.	dn.	− 0.15	***0.00***	− 0.03	0.55	− 0.06	0.21	− 0.23	0.52	− 0.08	0.83	− 0.49	0.15	− 0.08	0.83	− 0.59	0.07	0.02	0.96
med.	high	dn.	− 0.11	**0.01**	− 0.12	**0.01**	0.01	0.87	− 0.02	0.95	0.09	0.80	0.15	0.69	− 0.34	0.34	− 0.29	0.41	− 0.48	0.16
high	low	dn.	0.02	0.71	0.01	0.86	− 0.05	0.30	− 0.28	0.43	0.01	0.97	− 0.26	0.48	− 0.46	0.19	0.22	0.55	− 0.56	0.09
high	med.	dn.	− 0.12	**0.01**	− 0.13	***0.00***	− 0.09	**0.04**	0.07	0.84	0.15	0.69	− 0.56	0.09	− 0.29	0.42	− 0.44	0.20	− 0.23	0.52
high	high	dn.	− 0.05	0.31	− 0.09	**0.05**	− 0.05	0.23	− 0.61	0.06	0.37	0.29	0.01	0.99	− 0.22	0.54	0.10	0.79	− 0.36	0.30
low	low	up	− 0.06	0.21	0.01	0.85	0.14	**0.00**	− 0.01	0.99	0.41	0.24	− 0.20	0.58	0.70	**0.03**	0.24	0.51	0.48	0.16
low	med.	up	0.06	0.22	− 0.11	**0.02**	0.15	***0.00***	− 0.27	0.45	0.60	0.07	− 0.18	0.63	0.33	0.35	0.42	0.23	0.81	**0.00**
low	high	up	0.03	0.51	0.16	***0.00***	0.24	***0.00***	0.26	0.47	0.12	0.74	0.49	0.15	0.48	0.16	− 0.02	0.96	0.13	0.73
med.	low	up	− 0.05	0.32	− 0.06	0.16	0.00	0.98	0.14	0.70	− 0.09	0.81	0.40	0.25	0.47	0.17	0.26	0.47	0.83	**0.00**
med.	med.	up	0.10	**0.03**	0.12	**0.01**	0.12	**0.01**	− 0.31	0.38	0.23	0.53	0.65	**0.04**	− 0.05	0.88	0.41	0.24	0.32	0.37
med.	high	up	0.03	0.57	0.11	**0.02**	0.06	0.21	− 0.30	0.40	− 0.08	0.83	0.30	0.40	− 0.02	0.96	0.03	0.93	0.45	0.19
high	low	up	− 0.10	**0.03**	0.16	***0.00***	0.09	0.06	0.09	0.81	0.46	0.18	0.01	0.97	0.51	0.14	0.29	0.42	0.62	0.05
high	med.	up	− 0.01	0.90	0.10	**0.03**	− 0.05	0.30	− 0.19	0.60	− 0.19	0.60	0.07	0.85	− 0.16	0.65	0.27	0.45	0.54	0.11
high	high	up	0.02	0.73	0.11	**0.02**	− 0.02	0.65	− 0.32	0.41	0.15	0.70	0.53	0.14	− 0.13	0.75	− 0.49	0.19	− 0.16	0.68

## Discussion

### Quality control measures

Our prediction that PSC would show a positive relationship with mean change was met in all four experiments, indicating that participants understood the task and responded appropriately. All four experiments found a main effect of direction on the subjective change response, with higher responses to an increase in either stimulus frequency or intensity. This corresponds to findings in previous literature, e.g. loudness and duration of stimuli have been shown to be overestimated when stimuli are increasing in intensity^10,11^. This is likely the result of auditory *looming bias*: an increase in saliency to auditory stimuli with increasing intensity as an evolutionary response to approaching sounds^12,13^. Higher frequency sounds are often associated with sources of (or reporting of) threat, and therefore a similar bias seems intuitive.

### Bayesian and counter-Bayesian relationships between stimulus sequence precision and PSC

Our prediction that stimulus precision would positively relate to the behavioural response in a Bayesian response manner (Fig. 1Ci), as opposed to non-Bayesian (1Cii) or counter- Bayesian (1Ciii), was not met. All four experiments found main and/or interaction effects of precision, however, in many cases precision was inversely related to PSC (e.g. in Exp. 1 and Exp. 2 when mean change was low, Exp. 3 where mean change was medium, & Exp. 4 for all mean change conditions), and PSC response decreased with increased precision in a counter-Bayesian manner. This is unexpected and counter-intuitive, as more precise stimulus sequences would reduce noise in the data and therefore indicate more reliable information via increased signal-to-noise ratio. This paradoxical effect of precision potentially indicates the dissociability of informational precision of the sensory signals in the environment from encoded estimated precision within the brain, as an indicator of the reliability and importance of those signals. Put another way, many instances were observed where participants responded to less reliable sensory input in a manner that may imply it carried a higher salience or behavioural relevance.

Whilst in most instances, increased PSC with lower precision was associated with less accurate reporting of the direction of change (e.g. for downward intensity changes, and some instances of frequency changes)) or unaffected accuracy (e.g. most upward intensity change conditions), there were also more limited instances (notably for medium mean changes in frequency in Expt. 3) where lower precision was associated with both increased PSC and increased accuracy.

Interactions of mean change and precision indicated that the paradoxical effect of precision applies particularly to smaller absolute changes in stimulus statistics, whilst larger absolute changes more often showed Bayesian relationships, and intermediate changes precision-indifferent or mixed. This counter-Bayesian relationship was also more evident in intensity changes than in frequency changes. Interactions of precision and direction may reflect looming and related biases and indicate biases in direction of inferred change under uncertain conditions. Interactions of all three are a combination of the above factors. In the following paragraphs we consider why this might be the case and its potential relevance for our understanding of perceptual inference and its disorders.

A key consideration is whether participants were strongly influenced by individual extreme stimulus values, as opposed to the overall change between halves of the stimulus trials taking into account all stimuli. Using the values of a limited number of extreme stimuli might constitute a heuristic that is more efficient than integrating all sensory information. In principle, lower precision trials might favour increased perception of change via larger differences between individual stimuli. Our correlational analyses between maximal individual stimulus differences and PSC responses indicated that extreme stimuli within a trial did often influence PSC responses. This was the case for Exp. 2, which features small numbers of individual stimuli which varied in frequency. Conversely, such correlations with individual stimulus contrasts were not strongly evident where there were larger numbers of frequency-varying stimuli (Exp. 3), or where intensity was the varied parameter (Exp. 4). We consider several alternative or complementary explanations of this effect, below. Some of these would be mediated by extreme individual stimulus values, which only correlated with PSC ratings in certain experiments or stimulus conditions, and therefore different explanations might predominate in different situations.

There is a precedent for adding noise to sensory signals facilitating detection of changes, in a process termed *stochastic resonance*^14^. Whilst not identical, the effect we observe here could be analogous in terms of a mechanism to aid the detection of subtle changes in the sensory environment under conditions of additional noise.

A further explanation could be that greater variability between individual stimuli increases stimulus-driven attention, and/or requires greater listening effort, in effect increasing the effective precision of those stimuli in the brain^9^.

It is also possible that more variable (less precise) stimulus sequences reduce the extent of neuronal adaptation, leading to greater sensitivity to similar stimuli. This might have additional applicability to intensity changes, where rapid adaptation would be presumed to progressively reduce the perceived loudness of sounds within a trial. Listeners likely account for expected adaptation to some extent, therefore differences in actual adaptation on account of low precision might result in louder-than-expected sounds, thus be inferred as upward changes. Conversely, lower precision in experiments that vary frequency might result in individual outlier stimuli that are further from the mean, and therefore less attenuated by stimulus-specific adaptation, and therefore more influential in overall perceptual inference.

Another possibility relates to the number of stimuli needed to be integrated to form a reliable estimate of the stimulus mean, which will be a greater number for low precision, hence a longer time window over which to integrate them by assimilating prediction errors, resulting in an increased total integrated surprise over that period.

Finally, and particularly in the case of frequency changes where we saw the most correlations between PSC and extreme individual stimuli, it is possible that the added variability associated with low precision results in a greater number of individual stimuli that are relative outliers in the context of the entire experiment, and that over-weighting of the salience these rarer values might lead to greater influence on the processes of perceptual inference.

Exp. 3, which used a larger number of shorter stimuli, followed a slightly different results pattern, with fewer effects of precision. This could have been because larger stimulus numbers provide more complete indications of probability distributions, hence requiring fewer assumptions. Similarly, a larger number of stimuli occurring within a critical decision-making timeframe could lead to more accurate responses. Precision having less influence in the results pattern could also indicate a mixture of Bayesian and Counter-Bayesian effects in a different proportion for shorter stimuli.

### Behavioural accuracy and stimulus sequence precision

Clear relationships between size of mean change and direction discrimination indicates that participants understood the task and responded appropriately. Exp. 4, which varied stimulus intensity, found a main effect of precision, and both experiments found all interaction effects involving precision to be significant. In Exp. 4, downward intensity changes were detected more accurately with higher precision in a Bayesian response pattern, whilst upward intensity changes showed a non-Bayesian response pattern, except the smallest mean increase, where results instead indicated a bias from inferring downward changes towards upward ones, rather than altered accuracy per se.

However, notably, Exp. 3 contained the one instance where lower precision was associated with both greater PSC and improved direction discrimination accuracy. This result could be indicative of opposing mechanisms, e.g. that decreased precision aids performance (through one or more of the mechanisms suggested previously, e.g. stochastic resonance or increased salience), but this is specific to a certain difficulty of identification. i.e. If the mean change is high, the decision may be too easy for precision to have much of an effect on accuracy. If the mean change is low, the mechanism(s) that increases performance in low precision conditions may be insufficient to outweigh the increased noise. A recent study exploring how different cues elicit bias in perception similarly found contrasting effects of stimulus cues affecting perception at different levels of signal-to-noise ratio^15^. High signal-to-noise ratio biased participants’ responses away from recent stimuli, whereas low signal-to-noise ratio biased participants towards recent stimuli; after hearing high frequency stimuli participants were less likely (and slower) to report a high frequency test tone as high frequency when signal-to-noise ratio was high, whereas in low signal-to-noise ratio conditions participants who heard high frequency stimuli were more likely to rate a high frequency tone as high frequency.

Findings show the PSC response to be largely distinct from accuracy, suggesting it was not merely indicating ease of detection. Furthermore, increased perception of subjective change with low precision largely (with some notable exceptions) occurred in the same conditions where accuracy was lower. Under low precision conditions for intensity, a small decrease in mean intensity is reported as more surprising than larger decreases. As performance is close to chance level, we can infer that half the time participants are perceiving (or at least inferring) upwards intensity changes, which is consistent with both reported surprise and accuracy of change direction measures. This effect might only apply to low precision and small downward change, because this is the condition with the hardest to detect downward change, which is therefore most likely to be misinterpreted. Additionally, differences in intensity adaptation, and how these deviate from expected adaptation, between different precisions could lead to biases in both the size and direction of inferred change, which need not be consistent with each other.

### Wider implications of counter-Bayesian precision effects

It is important to highlight that this paradoxical effect of precision potentially indicates a situation where the brain may behave in a counter- or non-Bayes optimal manner. However, that does not mean that behaviour is sub-optimal, because the low signal-to-noise situations where we have detected counter-Bayesian behaviour may reflect the participants aiming to ‘optimize’ their behavior by keeping their options open and not overly relying on precision to guide their behavioral choices. Under low SNR conditions, precision may be register as ‘imprecise’ in the neural system. If there are instances where the neurotypical and healthy brain is already behaving in a Bayes-suboptimal manner in normal processes, this could be valuable in further understanding clinically disordered states associated with sub-optimal Bayesian inference, in which these biases might be quantitatively or qualitatively different. Such potentially relevant conditions include tinnitus^16,17^, chronic pain^18^, anxiety disorders^19^, autism^20^, hallucinations^21–23^ and psychosis^26–29^.

There is likely an evolutionary benefit to the over-caution of erring towards perceiving a larger change (or a change vs. no change) in uncertain conditions, as signals indicating potential threats might be largely obscured by noise in some situations, and a bias towards over-perception of potential threats might confer more benefits in more sensitive detection of true positives than detrimental consequences of false alarms. The findings of this study may suggest a mechanism to facilitate the detection of subtle signals in noisy environments.

### Limitations

This study is, to our knowledge, a first report of a counter-Bayesian phenomenon in human behaviour, which as noted appeared to be applied by the participants under certain low precision conditions whilst under other conditions Bayesian or other (e.g. precision-indifferent) responses were more parsimonious explanations of the results. We have aimed for the main interpretations to accurately reflect the general impression of results from the four sets of experiments, given the inherent variability that is often seen by running different experiments. Therefore, we have aimed to not rely on just a single experiment and therefore only interpret effects that were consistent across multiple experiments. However, we recognize that further variants and experiments could have been conducted. Therefore, although this study raises the possibility of counter-Bayesian approach being part of the basis of human behaviour, it is important to acknowledge some of the limitations of this study and how this hypothesis could be tested further with future work. To ensure that this study can provide a foundation for such future work, we highlight the limitations of this study for future work to be able to address, as follows.

With regards to how the participants were instructed, whilst we have implemented two different phrasings to capture PSC (how ‘Noticeable’ or ‘Surprising’). A more detailed characterisation of the subjective experience being reported would have been helpful, along with the impact of specific instruction or terminology. Moreover, our study design did not include a no change condition, or give participants the opportunity to report no change. Including these conditions and possibilities in a future study would be one way to mitigate any consequences of this. The present design might also potentially encourage ‘guessing’ a perceptual change response, rather than solely reporting what was directly experienced. Future studies might address such distinctions, for instance by obtaining confidence responses for each trial and including no-change conditions and giving participants a chance to report that they perceived no-change.

Although the key observations were replicated between these four studies, this paradoxical effect of counter-Bayesian responses to stimulus precision was observed in a limited parameter space, including low numbers of stimuli per sequence, and therefore it is important to understand how generalisable it is. Varying the signal-to-noise ratio more widely (i.e. greater variance between mean change and precision) would help explain how far this effect generalises. It is also worth noting that change always occurring at a fixed midpoint, which we specified to reduce additional sources of variance in the data adds an element of predictability to the trials that could influence responses. In future studies, randomizing the change point might remove any potential reliance on the midpoint being a cue for change and reduce potential bias.

We note that there are further aspects of the relationships between subjective surprise and stimulus precision still to explore, such as the exact extent of generalisability, and any learning effects over the course of an experiment. These could be examined in future studies. Additionally, it is presently unclear whether any of the paradoxical effects of precision also apply to ‘perceived size of change’, as they do to surprise as demonstrated here. Paradigms including points of subjective similarity (PSS), for instance, might help to establish this.

As studies were run online, it remains to be established how the results compare when experiments are performed under laboratory conditions. This would also provide the opportunity to correlate these behavioural findings with specific physiological responses. One potential explanation for the paradoxical effects of precision relates to increased attention or listening effort in lower precision conditions. This could be assessed in future laboratory-based studies using physiological measures such as pupil size or by implementing these experiments with task-irrelevant attentional-manipulation conditions.

Finally, this study was only conducted in humans. Therefore, our human study raises the important question of whether counter-Bayesian behaviour may be seen in nonhuman animals, and if so in which species besides humans. If other species are found to also show counter-Bayesian behaviour under certain conditions, and there may be related examples from the reinforcement learning literature where human and nonhuman animals take non-optimal reward maximization strategies^30^, the neurobiological mechanisms of such behaviour could be studied not just with the approaches available to humans but also with the neuronal circuit-cracking tools available in animal models of human behaviour.

## Conclusions

We have shown that increasing precision of stimulus sequences does not necessarily make otherwise equivalent sensory changes more surprising or easier to detect, and in some cases that it has the opposite effect. This paradoxical effect of precision indicates the brain potentially behaving in a counter-Bayesian manner, with inherent biases towards over-inferring small changes in noisy conditions, and towards detecting small increases, mainly in intensity. These observations are important for our fundamental understanding of perceptual inference, studying neurobiological mechanisms, and advancing understanding clinical conditions where Bayesian and counter-Bayesian behaviour is disordered.

## Methods

### Subjects

All studies were run online. Subjects for Experiments 1 and 2 were collected online through questionnaire/experiment sharing Facebook groups. Subjects for experiments 3 and 4 were collected through www.prolific.co and were paid £5 for participation. Experiments 1, 2, 3, and 4, had 73, 48, 93, and 70 participants respectively. Informed consent was received from each participant, no personally identifiable information was taken, and they had the option to withdraw at any time. We did not include exclusion criteria to remove underperforming participants. Pilot tasks showed all participants to use a full range of responses and did not press the same button in more than 95% of trials. For the full experiments, a visual check of each participant’s mean responses per condition did not identify any instances of implausible or unvarying results, and therefore we did not feel exclusion criteria necessary.

### Procedure

Each study followed the same overall procedure; differences in stimulus parameters, stimulus values, and experimental design are highlighted in Table 2. Each experiment consisted of 180 trials: 10 exemplars of each of the 18 different combinations of stimulus properties (low, medium, high precision; low, medium, high mean change; mean change direction upward or downward – see Fig. 1). The 10 unique exemplars per combination were the same across all subjects, but the order of stimuli was randomised for each subject. What differed between experiments was the values of these parameters, the instruction given to participants, the number and duration of stimuli, and/or whether stimulus frequency or intensity was the varied parameter. The rationale behind these alterations in the experiments were to establish generalisability through altered phrasing (Exp. 2), altered timescale (Exp. 3), and altered modality (Exp. 4).

Exp. 4 featured intensity changes, and first required subjects to set a comfortable maximum intensity level prior to starting the experiment, with stimulus intensities set relative to (always below 90% of) this reference (maximal) value. All subjects performed four practise trials to familiarise them with the task, before completing all 180 trials continuously without breaks, taking approximately 20 min. Each trial consisted of a series of pure tones being played, with a change in mean frequency (or intensity, see Table 2) occurring exactly half-way through each trial. Participants were prompted, after each trial, to give a behavioural response on a four-point Likert scale; how “surprising” (or “noticeable”, see Table 2) the change was e.g. “How noticeable was the change between the first half and the second half of the stimuli?” responding 1–4 on the keyboard. 1 = ‘Not at all noticeable’, 2 = ‘Slightly noticeable’, 3 = ‘Quite noticeable’, 4 = ‘Very noticeable’. In Experiments 3 and 4, subjects gave an additional rating after each trial to indicate whether they perceived the change to be upward or downward.

### Stimuli

Stimuli were generated in MATLAB (R2021b, version 9.11.0.1769968). Each trial of 10 (or 30) auditory stimuli varied in frequency (or intensity), drawn from a Gaussian distribution with a mean that changed halfway through each trial and a fixed precision (inverse of variance). The main stimulus properties that were altered for each trial were: mean change (octaves or dB), precision (inverse of variance, measured in octaves^−1^ or dB^−1^) and the direction of the mean change (up or down). Mean change and precision values for each experiment were designated either ‘high’, ‘medium’, or ‘low’ (see Fig. 1), the values for these can be seen in Table 2. Before the practise sessions, participants were played representative test sounds, and instructed to set their system volume to ensure all stimuli were both clearly audible and at a comfortable loudness.

The starting parameters were arrived at through informal preliminary testing at the point of development. These stimulus parameters were judged to best represent our interest in the computations of perceptual inference. Responses to mean change size reflect inferences about both the perception of the size of a change and also surprise in the face of overall stimulus change. Including precision allowed us to factor in the perception of, and response to, stimuli with different levels of signal to noise ratio. Direction allowed us to understand and account for bias relating to increases or decreases in frequency or intensity. The stimulus values were also determined through informal piloting and were chosen to span a range of subtle to obvious changes, whilst balancing the input of both the mean change and the precision.

**Table 2:** Differences in stimulus parameters and experimental design between experiments.

	Experiment 1	Experiment 2	Experiment 3	Experiment 4
Varied parameter	Frequency	Frequency	Frequency	Intensity
Stimuli per trial	10	10	20	10
Stimulus duration (s)	0.3	0.3	0.08	0.3
Trial Length (s)	3.36	3.36	2.36	3.36
Subjective change response	‘Noticeability’	‘Surprise’	‘Surprise’	‘Surprise’
Performance measure	N/A	N/A	Change direction	Change direction
Precision (low, medium, high) (oct^−1^/dB^−1^)	16, 32, 64	16, 32, 64	16, 32, 64	1/32, 1/8, 1/2
Mean change (low, medium, high) (oct/dB)	0.25, 0.5, 1	0.25, 0.5, 1	0.25, 0.5, 1	2.5, 5, 10

### Data analysis

Data analysis was conducted in RStudio (R version 4.2.2 (2022-10-31 ucrt)). As an initial step, each subject’s responses were averaged across trials within each of the 18 stimulus conditions, and these subject averages formed the basis of statistical testing. For each experiment, normality of the distribution of data was visually assessed using the qqplot function, and a three-way repeated measures ANOVA with full interaction terms was used to determine if perceived salience of a change (reported noticeability or surprise) related to the size of the change in stimulus mean between the first and second half of the stimuli (mean change), the inverse variance of the stimuli (precision), and whether the stimulus change was an increase or decrease in frequency (direction), or in intensity in Exp. 4. For experiments 3 and 4, a three-way repeated measures ANOVA was run to determine if accuracy of response (proportion of correct responses indicating whether the mean change went up or down in frequency) related to the size of the change in stimulus mean between the first and second half of the stimuli (mean change), the inverse variance of the stimuli (precision), and whether the stimuli change was an increase or decrease in frequency or intensity (direction). For all ANOVAs, Mauchly’s Test for Sphericity was used with Greenhouse-Geisser corrections if assumptions of sphericity were not met. Post hoc tests were run for each significant interaction effect, first by breaking the three-way interaction into two-way interactions, and then by comparing simple main effects for each significant two-way model. Finally, pairwise comparisons were run for each significant main effect for precision. At each stage, Bonferroni adjustment was used for multiple comparisons.

We also sought to establish the contribution that individual extreme stimulus values might make to subjective ratings of change. To do this, for each of Exp. 2–4 (Exp. 1’s original stimulus data were lost due to a storage error, but the stimuli were equivalent to those in Exp. 2), we calculated the following four metrics for every exemplar (of which there were 10 per condition): (1) Midpoint change, i.e. value (in oct or dB) of Stimulus 6 minus Stimulus 5 (for Exp. 2 and 3) or Stimulus 16 minus Stimulus 15 (Exp. 4); (2) Minimum value in second half minus maximum value in first half; (3) Maximum value in second half minus minimum value in the first half; (4) Maximum value anywhere in the trial minus minimum value anywhere in the trial. For each metric, a Spearman correlation coefficient was calculated, across the 10 exemplars, between the metric’s value and the mean PSC rating for that exemplar across all participants. We do not report the results of metric 4, as it did not capture any correlations not already better explained by metrics 2 and 3. A total of 54 correlation coefficients were obtained per experiment (18 stimulus conditions, 3 metrics). As this was both an exploratory analysis and a control analysis, we presented the results in both uncorrected and false discovery rate (FDR) corrected forms (in the latter case, for 54 comparisons). FDR correction used the Benjamini-Hochberg method.

## Data Availability

The data that support the findings of this study; and the code used to compile data, perform analysis, and create figures; are openly available in the open science framework at https://osf.io/b7p8n/?view_only=a27c6e838804406599458bde75cb1822.
